# Integrated Analysis of mRNA Expression, CpG Island Methylation, and Polymorphisms in the *MITF* Gene in Ducks (*Anas platyrhynchos*)

**DOI:** 10.1155/2019/8512467

**Published:** 2019-09-23

**Authors:** Ruiyi Lin, Weimin Lin, Shiye Zhou, Qiaohui Chen, Jiahua Pan, Yuanxin Miao, Mengwen Zhang, Zhongbin Huang, Tianfang Xiao

**Affiliations:** ^1^College of Animal Science, Fujian Agriculture and Forestry University, Fuzhou 350002, China; ^2^Research Center of Waterfowl Genetic Resource Protection of Shishi in Fujian Province, Quanzhou 362700, China; ^3^Jingchu University of Technology, Jingmen 448000, China

## Abstract

Microphthalmia-associated transcription factor (*MITF*) is a key regulator for the development and function of melanocytes in skin, eye, and plumage pigmentations. Thus, the *MITF* was selected as a candidate gene associated with plumage coloration in ducks. This study analyzed the mRNA expression, promoter methylation, and polymorphisms in the *MITF* gene in ducks with different plumage colors (Putian Black, Putian White, Liancheng White, and Longsheng Jade-green). No expression of the *MITF* melanin-specific isoform (*MITF-M*) was detected in white feather bulbs. By contrast, the mRNA expression levels of *MITF-M* were high in black feather bulbs. Bioinformatics analysis showed that two CpG islands were present in the promoter region of the *MITF* gene. The methylation level of the second CpG island was significantly lower in black feather bulbs than in white feather bulbs. However, the methylation level of the first CpG island was not different among the feather bulbs with various colors except Liancheng White feather bulbs. The methylation status of the whole CpG island significantly and negatively correlated with the mRNA expression of *MITF-M* (*P* < 0.05). Furthermore, four novel SNPs (single nucleotide polymorphisms) were identified in the 5′UTR, exon 4, intron 7, and intron 8 of the *MITF* gene. Allele T in g.39807T>G and allele G in g.40862G>A were the predominant alleles only found in Putian White, whereas the variant A allele in g.32813G>A exhibited a high allele frequency in Liancheng White. Collectively, these results contributed to the understanding of the function of the *MITF* gene in duck plumage coloration.

## 1. Introduction

Plumage color is an important characteristic of duck breed. In commercial duck meat processing, white plumage ducks have an advantage over black ones because the carcasses of white plumage ducks after plucking are preferred by consumers. Plumage color variation is mainly determined by two kinds of melanin, namely, eumelanin and pheomelanin [1]. In the process of melanin biosynthesis, tyrosinase, encoded by the *TYR* gene, is the rate-limiting enzyme affecting the production of melanin pigment [2] and determines which type of melanin can be synthesized [3]. The transcription of *TYR* family genes is activated by *MITF* (microphthalmia-associated transcription factor) through binding to M-box in the upstream regulatory region [4]. The gene encoding *MITF* is a member of the Myc superfamily, which is part of the basic helix-loop-helix-leucine zipper family [5, 6]. Kawasaki et al. [7] reported that *Mitf* is involved in the regulation of melanosome transport and the level of dendricity in melanophores using *Xenopus laevis* as a model system. A high *Mitf* expression is observed in Silky Fowl, which is a natural mutant with hyperpigmentation in various internal tissues [8].

*MITF* mutations can lead to coat color depigmentation, white spotting, or complete pigmentation loss in several vertebrate species [9, 10]. In humans, *MITF* gene mutations are responsible for Waardenburg syndrome type II, which is characterized by sensorineural hearing loss and depigmented patches of skin and hair [11]. *Mitf*^*mi-bw*^ mutant mice harbor an insertion of a long interspersed element-1 in intron 3, severely affecting the expression of functional *Mitf-M* and resulting in a complete black-eyed white phenotype with severe hearing loss in the homozygous genotype [12]. Subsequent studies have also reported that variants in *MITF* are associated with white spotting in dogs [13, 14], piebaldism in cattle [15], white markings in Spanish horses [16], and white coat coloring in American Standardbred foals [17]. In addition, genome-wide analysis has revealed that *MITF* is regarded as a strong candidate for white spotting patterns in Chinese domestic pigs [18].

*MITF* also plays a critical role in plumage coloration in poultry. The causal mutation *B* in the *MITF* gene, which results from a premature stop codon caused by a 2 bp deletion in exon 11, is responsible for the “silver” plumage color in Japanese quail and implicated in its growth and body composition [19]. On the other hand, a single nucleotide polymorphism (SNP) in the *MITF* gene has been significantly associated with the white plumage trait of Zhedong White geese [20]. In ducks, the differential expression and polymorphisms of the *MITF* gene have been proposed to be associated with different plumage color phenotypes [21, 22]. Moreover, whole-genome resequencing has revealed that white plumage in duck is a result of the selection at the *MITF* locus [23]. Zhou et al. [24] identified an ∼6.6 kb insertion between exon 1 M and exon 2 in *MITF* that accounts for the white feathers of Pekin duck.

Although some polymorphisms of the *MITF* gene in ducks have been investigated, the mechanism that controls the variation in plumage color has yet to be explored. In the present study, the *MITF* gene was investigated to elucidate its characteristics by analyzing its mRNA expression, promoter methylation, and plumage color-related SNPs in Chinese native ducks.

## 2. Materials and Methods

### 2.1. Ethics Statement

All animal procedures were approved by the Experimental Animal Care and Use Committee of Fujian Agriculture and Forestry University (FAFU2013-0012) according to the Regulations for the Administration of Affairs Concerning Experimental Animals (Ministry of Science and Technology, China, revised in July 2013).

### 2.2. Animals and Tissues

**Figure 1:**
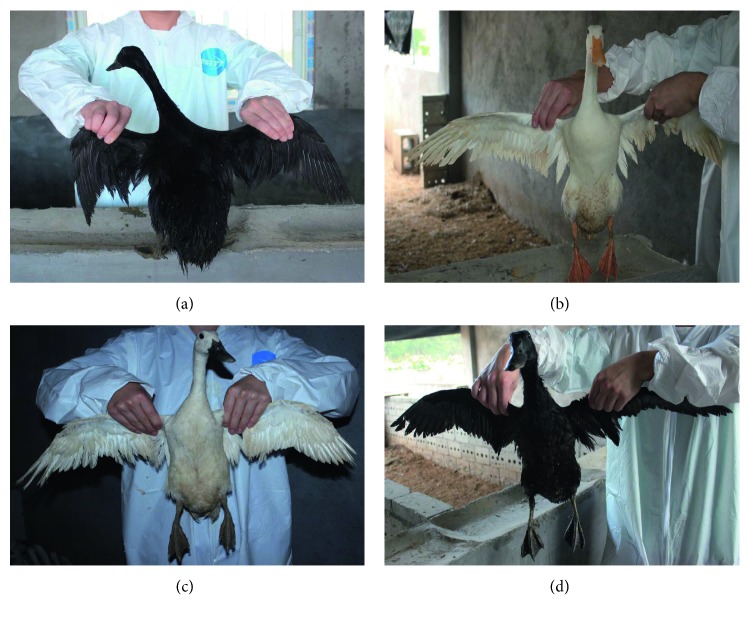
Ducks with different plumage colors. (a) Putian Black duck (PTB); (b) Putian White duck (PTW); (c) Liancheng White duck (LC); (d) Longsheng Jade-green duck (LS). PTB duck is the only indigenous breed with black plumage on the whole body. It has black beaks and webbed feet. PTW duck has white plumage, yellow beaks, and webbed feet. LC duck has white plumage, black beaks, and webbed feet. LS duck has glossy black feathers, black beaks, and webbed feet. Male duck has a malachite green head.

Putian Black (PTB), Putian White (PTW), Liancheng White (LC), and Longsheng Jade-green (LS) ducks are native breeds in China (Figure 1). All of them were raised in the National Waterfowl Germplasm Resource Pool (Shishi, Fujian, China). Blood samples were collected from 432 ducks (112 PTB ducks, 107 PTW ducks, 107 LC ducks, and 106 LS ducks) and stored at −20°C. The feather bulbs obtained from four ducks of each breed (a total of 16 samples) were immediately frozen in liquid nitrogen and stored at −80°C.

### 2.3. Bioinformatics Analysis

BLAST was used to search the duck *MITF* promoter region sequence based on the duck genome data (BGI_duck_1.0) and chicken *MITF* promoter sequence (GenBank Accession no. FJ196874). The CpG island of the duck *MITF* promoter was predicted by MethPrimer (http://www.urogene.org/cgi-bin/methprimer/methprimer.cgi). AliBaba2.1 was used to identify putative transcription factor-binding sites (TFBS) in the CpG island (http://www.gene-regulation.com/pub/programs/alibaba2/index.html).

### 2.4. DNA and Total RNA Extraction

DNA samples were extracted from the blood and feather bulb specimens via a standard phenol-chloroform method and then stored at −20°C until further use. Total RNA was extracted from feather bulb specimens using TRIzol reagent (Invitrogen, Carlsbad, USA) according to the manufacturer's instructions. The concentrations of isolated DNA and RNA were determined using a NanoDrop 2000 (Thermo Fisher Scientific, Leicester, UK), and denaturing gel electrophoresis was conducted to assess the quality of RNA.

### 2.5. Methylation Analysis of CpG Islands

Genomic DNA from feather bulbs was treated with bisulfite using the EZ DNA Methylation-Gold Kit™ (Zymo Research, Irvine, CA, USA) according to the manufacturer's protocols. PCR was performed in a total volume of 50 *μ*L, containing 150 ng of DNA, 5 *μ*L of PCR buffer (10x), 150 *μ*M dNTPs, 3 *μ*M of each primer, 1 *μ*L of Taq DNA polymerase (5 U/*μ*L), and double-distilled H_2_O up to 50 *μ*L. The following reaction conditions were used: 94°C for 5 min; 30 cycles of 94°C for 30 s, 58.5/60.5°C for 30 s (Table 1), and 72°C for 25 s/1 min; and a final extension at 72°C for 10 min. The PCR products were examined by 1.5% agarose gel electrophoresis and purified using a MiniBEST Agarose Gel DNA Extraction Kit (TaKaRa, Dalian, China). The expected fragment was inserted into the pMD18-T vector, and recombinant clones were used to transform Trans5*α* Chemically Competent Cells (TransGen Biotech, Beijing, China). The positive recombinant clones were selected on LB medium containing 60 *μ*g/mL ampicillin and confirmed by PCR. Ten to fifteen positive recombinant clones selected from each individual were sequenced to identify the mutation and methylation sites. The *MITF* normal primer (Table 1) was designed for unmethylated DNA amplification.

### 2.6. Expression of *TYR* and *MITF* in Duck Feather Bulbs

*TYR*, as a marker gene of melanocyte [25], was used to confirm the feather bulbs with different colors. *MITF* consists of at least five isoforms with a distinct amino-terminal in humans and mice, and the mRNA expression of *MITF-M* is exclusively expressed in melanocytes and pigmented melanoma cells [10, 26]. The specific primer of *MITF-M* was designed according to Li et al. [21] on the basis of the alternative 5′exon.

Total RNA was transcribed into cDNA using a PrimeScript™ RT Reagent Kit with gDNA Eraser (TaKaRa, Dalian, China), containing oligo (dT), random primers, RT enzyme, and gDNA Eraser. Quantitative real-time PCR (QPCR) was performed using the ABI Prism 7500 sequence-detection system (Applied Biosystems, Foster City, CA, USA) with SYBR Green Real-time PCR Master Mix (Toyobo, Osaka, Japan) following the manufacturer's instructions. All PCR assays were performed in triplicate, and *β-actin* was used as an internal control. The fold change in relative gene expression was calculated using the standard 2^−ΔΔCt^ method [27]. The ΔCt of the LS1 sample was arbitrarily set to 1 for the relative quantification of the expression levels of genes in the other groups (ΔΔCt = ΔCt of each group −ΔCt of the LS1 group in each experiment).

### 2.7. Polymorphism Detection of Duck *MITF* Gene

Six pairs of primer were designed on the basis of the genomic sequence of the duck *MITF* gene (GenBank Accession no. KY114890) to scan the polymorphisms of this gene ([Supplementary-material supplementary-material-1]). The PCR assay contained 50 ng of genomic DNA, 2 *μ*L of PCR buffer (10x), 1 unit of Taq DNA polymerase, 100 *μ*M dNTPs, 1 *μ*M of each primer, and double-distilled H_2_O up to 20 *μ*L. Five randomly selected DNA samples from each duck breed (a total of 20 samples) were used as PCR templates. The PCR conditions were as follows: 5 min initial denaturation at 94°C; 35 cycles of 94°C for 10 s, 57.6/60.5°C for 30 s (Table 1), and 72°C for 1 min; and a final extension step at 72°C for 10 min. PCR products were detected through electrophoresis in 1.5% agarose gels and sequenced by a commercial service (Sangon, Shanghai, China).

Specific primers (listed in Table 1) were designed to detect four SNPs in a large population. The PCR conditions were the same as those previously described. For PCR-RFLP (polymerase chain reaction-restriction fragment length polymorphism), 6 *μ*L of PCR products was digested with 2 units of *Hae*II, *Hae*III, *Apo*I, and *Hin*fI (NEB, Ipswich, Massachusetts, USA) for 4 h at 37°C. The enzyme-digested products were separated by electrophoresis on a 2% agarose gel with GelRed (Biotium, CA, USA).

### 2.8. Statistical Analysis

The sequencing results of methylation analysis were examined using QUMA (http://quma.cdb.riken.jp/) to calculate the methylation rate of CpG sites. The correlation between methylation levels and mRNA expression was analyzed by Pearson's correlation [28].

PIC (polymorphic information content) was calculated using the following formula:

(1) $$\begin{array}{c} \text{PIC}=1-\overset{n}{\underset{i=1}{\sum }}P_{i}^{2}-\overset{n-1}{\underset{i=1}{\sum }}\overset{n}{\underset{j=i+1}{\sum }}2P_{i}^{2}P_{j}^{2}, \end{array}$$

where *n* is the number of alleles at one locus and *P*_*i*_ and *P*_*j*_ are the frequencies of the *i*th and *j*th alleles at one locus, respectively, and *j* = *i* + 1.

## 3. Results

### 3.1. Expression of *TYR* and *MITF* Genes in Duck Feather Bulbs

**Figure 2:**
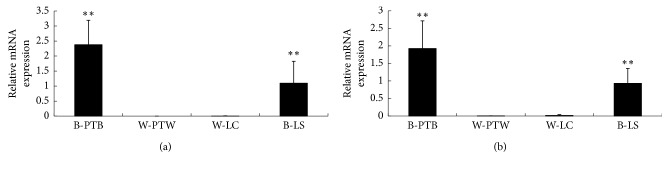
Gene expression in feather bulbs as determined by qPCR. B-PTB, black feather bulb from Putian Black duck; W-PTW, white feather bulb from Putian White duck; W-LC, white feather bulb from Liancheng White duck; B-LS, black feather bulb from Longsheng Jade-green duck. All assays were repeated at least three times, and data are shown as mean ± standard error (*n* = 4) from one representative experiment. The expression of each gene was normalized to *β-actin*. The asterisks (^*∗∗*^) indicate significant differences (*P* < 0.01). (a) *TYR* gene. (b) *MITF* gene.

The *TYR* expression was detected to further confirm the feather bulbs with different colors. The expression pattern of the marker gene was consistent with the difference in plumage color. Nearly no mRNA expression of *TYR* was observed in white feather bulbs (Figure 2(a)). It has been demonstrated that *MITF* is involved in pigmentation in several species; however, whether *MITF* is implicated in the feather coloration of Chinese native ducks is still unknown. To investigate that, we analyzed the expression profile of *MITF-M* isoform. Similarly, the *MITF-M* isoform was also not expressed in white feather bulbs (Figure 2(b)), whereas the B isoform of the *MITF* gene was expressed in both black and white feather bulbs (data not shown). These findings indicated that the melanogenesis pathway is implicated in the plumage color in ducks.

### 3.2. Bioinformatics Analysis of Duck *MITF* Promoter

DNA methylation occurs mainly in the CpG island-rich promoter region, and the sequence of the duck *MITF* promoter region needs to be clear in this study firstly. On the basis of the duck genome data (BGI_duck_1.0) and chicken *MITF* promoter sequence (GenBank Accession no. FJ196874), the sequence of the duck *MITF* promoter region was predicted through BLAST. The CpG island was analyzed using MethPrimer online software. Two CpG islands were identified in the 5′upstream region of the *MITF* gene ([Supplementary-material supplementary-material-1]). The CpG islands were located in the regions from −1762 to −1661 bp and from −1597 to −1467 bp containing 20 CpG dinucleotides. Several putative TFBS, including Sp1, AP-1, CREB, CPE_bind, Oct-1, MyoD, and p40x, were identified in the CpG islands. Two pairs of primer were designed on the basis of the location of the CpG islands and used for the amplification of two fragments containing the CpG island regions (Table 1).

### 3.3. Methylation Status of Duck *MITF* Promoter

**Figure 3:**
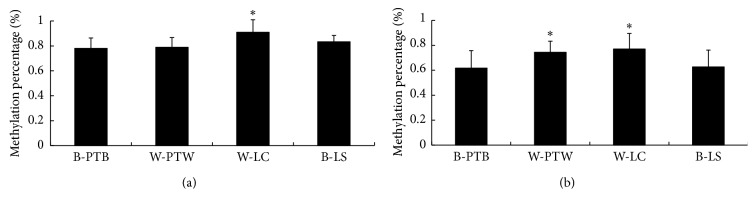
Methylation of CpG island in feather bulbs. B-PTB, black feather bulb from Putian Black duck; W-PTW, white feather bulb from Putian White duck; W-LC, white feather bulb from Liancheng White duck; B-LS, black feather bulb from Longsheng Jade-green duck. Each individual was sequenced at least 10 times to identify the methylation sites, and data are shown as mean ± standard error (*n* = 4) from one representative experiment. The asterisk (^*∗*^) indicates a significant difference (*P* < 0.05). (a) First CpG island. (b) Second CpG island.

DNA methylation is a major epigenetic modification that regulates gene expression. To analyze the influence of promoter methylation on the expression of the duck *MITF* gene, the methylation status of the duck *MITF* promoter was measured. Bisulfite sequencing PCR (BSP) was performed to amplify the CpG islands. The PCR products of normal and bisulfite-treated DNA were cloned and sequenced. The sequencing results were submitted to QUMA (http://quma.cdb.riken.jp/) for further analysis. The average methylation rate of the CpG sites was 75.07%, suggesting that the CpG islands in the duck *MITF* promoter had a high degree of methylation. As shown in Figure 3(a), the methylation status of the first CpG island was not significantly different among the feather bulbs of PTB, PTW, and LS. However, the degree of methylation was significantly higher in the white feather bulbs of LC than that in others (*P* < 0.05, Figure 3(a)). In the second CpG island, the methylation levels differed dramatically in the white and black feather bulbs (*P* < 0.05, Figure 3(b)).

Pearson correlation analysis (Table 2) showed that the methylation status of the whole CpG island was negatively correlated with the mRNA expression of *MITF-M* (*P* < 0.05), and significant correlation coefficients were obtained for CpG_4 and CpG_6 in CpG island 2 (*P* < 0.01).

### 3.4. Identification and Analysis of Polymorphic Loci in Duck *MITF* Gene

**Figure 4:**
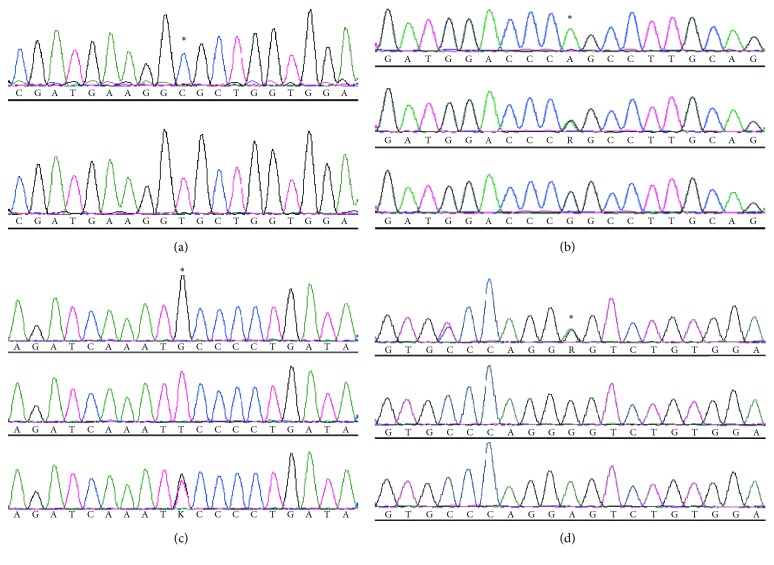
Four previously unreported SNPs in the duck *MITF* gene. (a) g.312C>T site; (b) g.32813G>A site; (c) g.39807T>G site; (d) g.40862G>A site. Five individuals from each breed (*N* = 20) were randomly selected for Sanger sequencing. Asterisk showed mutation sites. The SNPs were mainly observed in PTB and LS ducks according to the reference sequence of Peking duck.

To analyze the correlation between SNPs of *MITF* gene and color variants in ducks, we screened polymorphisms within whole region of the duck *MITF* gene through direct sequencing in twenty individuals randomly selected from the four duck breeds. We found four novel SNPs (g.312C>T, g.32813G>A, g.39807T>G, and g.40862G>A) according to the reference sequence of Peking duck. These SNPs, located in the 5′UTR, exon 4, intron 7, and intron 8, respectively (Figure 4), were mainly observed in PTB and LS ducks.

PCR-RFLP was used to detect the four SNPs in a large population ([Supplementary-material supplementary-material-1]). The genotype and allele frequencies of the identified SNPs of the *MITF* gene from four duck populations are shown in Table 3. Loci polymorphism can be considered high, medium, or low if PIC > 0.5, PIC > 0.25, or PIC < 0.25, respectively [29]. In the present study, except for PTB and LS in g.312C>T and PTB in g.32813G>A achieving moderate polymorphism, the others achieved low polymorphism. Table 3 shows that allele T in g.39807T>G and allele G in g.40862G>A were the predominant alleles and found exclusively in PTW and that the variant A allele in g.32813G>A exhibited a high allele frequency in LC.

The four duck populations can be categorized into group 1 (PTW duck) and group 2 (PTB, LS, and LC ducks) based on the genotype and allele frequencies of g.39807T>G and g.40862G>A in the *MITF* gene. Even though PTW and LC ducks both have white plumage, the beaks and webbed feet of PTW ducks are yellow, whereas those of LC ducks are black, similar to those of PTB and LS ducks (Figure 1). Here, the correlation analysis also showed that these two loci (g.39807T>G and g.40862G>A) were significantly associated with beak and webbed foot colors (*P* < 0.01).

## 4. Discussion

China has a rich genetic resource of domestic ducks, which are a source of meat, eggs, and feathers [30]. The color of duck plumage is a breed characteristic. However, some ducks with plumage color variation occur in the breeding process, especially in crossbreeding [31]. To obtain homogeneous plumage color ducks, breeders should have an enhanced understanding of plumage color genetics.

Differences in the melanin-based coloration of feathers are caused by the relative content and distribution of eumelanin and pheomelanin produced in melanocytes [32]. *MITF* has emerged as an essential regulator not only for melanocyte development, proliferation, and survival but also for the expression of enzymes ensuring melanin production [33]. In the present study, almost no mRNA expression of the *MITF-M* isoform was observed in white feather bulbs regardless of duck breed. In addition, reduced *TYR* expression in white feather bulbs might be attributed to the altered *MITF-M* expression. Thus, melanocyte development might be defective in white feather bulbs.

The sequence and methylation status of the *MITF* promoter region were analyzed to explore the regulatory mechanism of *MITF-M* expression in duck feather bulbs. Two CpG islands were predicted to be in the *MITF* promoter, and the methylation level of the second CpG island was significantly higher in white feather bulbs than in black feather bulbs. Pearson correlation analysis also showed that the methylation of the CpG islands was negatively correlated with *MITF-M* expression. These findings indicated that *MITF-M* expression is regulated by DNA methylation, which was consistent with the results reported by Lauss et al. [34]. Previous studies confirmed that variants of the *MITF* promoter are associated with unpigmented phenotypes in horses and dogs [35, 36]. The present study found that the methylation level of the *MITF* promoter was associated with duck plumage coloration. To the best of our knowledge, our study is the first to report the potential epigenetic mechanisms to explain the variation in duck plumage coloration [37]. However, further experiments are needed to study the mechanism of methylation differences in the white feather bulbs of PTW and LC ducks.

In addition, *MITF* was subjected to mutation screening to investigate the association of *MITF* gene with duck plumage colors. The comparison of *MITF* gene among the four Chinese native duck breeds identified four novel mutations (g.312C>T, g.32813G>A, g.39807T>G, and g.40862G>A) located in the 5′UTR, exon 4, intron 7, and intron 8, respectively. *MITF* polymorphisms in this study differed from those in other studies [22–24, 38] because of the different duck breeds. It is also worth mentioning that the *MITF* gene is large (KY114890 covers approximately 48.6 kb). Thus, the partial resequencing performed in this study might possibly miss other putative mutations.

Interestingly, the distributions of the *MITF* g.32813G>A, g.39807T>G, and g.40862G>A polymorphisms in the white plumage duck population were different from those in LC and PTW ducks. LC and PTW are two white plumage duck varieties. LC ducks have black beaks and webbed feet, whereas PTW ducks have yellow beaks and webbed feet. Considering the similarity of appearance between PTW and Cherry Valley ducks, the polymorphism of these three sites was further detected in Cherry Valley ducks. As expected, the genotype distribution of these three loci in Cherry Valley ducks was identical to that of PTW ducks. These three SNP variations might not only cause the differences in plumage color observed in this test group, but also be associated with the differences in the color of beaks and webbed feet.

As a complex trait, plumage color is determined by a complex pathway system and multiple interactive patterns, suggesting that the molecular mechanism of plumage color formation needs further investigation [39]. Our results provided novel information for elucidating the *MITF* function in plumage color variations in ducks.

## 5. Conclusion

On the basis of the results of this study, we speculated that the differentially methylated *MITF* promoter is related to *MITF-M* expression and affects the plumage color phenotypes in ducks. The variations of duck plumage color are not only affected by *MITF-M* mRNA expression, but also may be related to the genetic variants of the *MITF* gene.

## Figures and Tables

**Table 1:** Primer pairs used for PCR amplification.

Primer name	Primer sequence (5′-3′)	Product size (bp)	Tm (°C)
PCR-*Hae*II-RFLP	F: TATGAGTGCCTGCTGCTACCT	485	59.5
	R: GTTGATGGCAGGTGTCTGG		

PCR-*Hae*III-RFLP	F: TTGTACGGGCACCGATAT	457	52.6
	R: CTTGAGGAGCAGCGATTA		

PCR-*Apo*I-RFLP	F: CCTACAGAGTCAGAAGCAAGAG	329	57.2
	R: TTCCAAGAAGTCCAGATAAACAG		

PCR-*Hin*fI-RFLP	F: GCCACGTCAACAGTCCCACA	540	56.5
	R: GGCGGTCACTCACAGCATCAG		

CpG island normal primer	F: TTCCCGGCATCTGAACAAAG	983	60.5
	R: TCTCAACAGCAAAGGCACCC		

CpG island 1 BSP primer	F: GGTTGTTTTTAAAGTGTAATTTTGT	256	58.5
	R: AAACTCCCCTTAATATTTCATCCTC		

CpG island 2 BSP primer	F: AGAGGATGAAATATTAAGGGGAGTT	222	60.5
	R: ACCTACCTACCAAAAAACTCATTTC		

*TYR*	F: TTACATGGTCCCCTTTATTC	182	60
	R: CAATCACAGCTGCACCAACC		

*MITF*	F: CCCAGTTCATGCAGCAGAGAGT	268	60
	R: CCAGGCGGCATGACATGATCAC		

*β-Actin*	F: AACTGGGATGACATGGAGAAGA	189	60
	R: ATGGCTGGGGTGTTGAAGGT		

*Note*. RFLP, restriction fragment length polymorphism; BSP, bisulfite sequencing PCR; Tm, melting temperature.

**Table 2:** The correlation analysis between methylation level and mRNA expression in the *MITF* gene.

CpG island	CpG site	Correlation coefficient	*P* value
CpG island 1	CpG_all	−0.460	0.270
	CpG_1	−0.830	0.085
	CpG_2	−0.880	0.060
	CpG_3	0.576	0.212
	CpG_4	−0.004	0.498
	CpG_5	0.061	0.469
	CpG_6	0.135	0.432
	CpG_7	—	—
	CpG_8	−0.493	0.254
	CpG_9	−0.155	0.422
	CpG_10	—	—

CpG island 2	CpG_all	−0.910	0.045
	CpG_1	−0.109	0.445
	CpG_2	−0.661	0.169
	CpG_3	−0.269	0.366
	CpG_4	−0.996	0.002
	CpG_5	−0.714	0.143
	CpG_6	−0.996	0.002
	CpG_7	−0.661	0.170
	CpG_8	−0.867	0.066
	CpG_9	−0.753	0.124
	CpG_10	0.880	0.060

CpG island	CpG_all	−0.905	0.047

**Table 3:** Genotype distribution and genetic polymorphisms of the duck *MITF* gene.

SNP position	Breed	Genotype frequency (number of birds)			Allele frequency		PIC
		CC	CT	TT	C	T	
g.312C>T	PTB	0.35 (39)	0.54 (61)	0.11 (12)	0.621	0.379	0.360
	PTW	0.99 (106)	0.01 (1)	0 (0)	0.995	0.005	0.009
	LC	0.87 (93)	0.13 (14)	0 (0)	0.935	0.065	0.115
	LS	0.44 (47)	0.46 (49)	0.09 (10)	0.675	0.325	0.343

g.32813G>A		GG	GA	AA	G	A	
	PTB	0.36 (40)	0.51 (57)	0.13 (15)	0.612	0.388	0.362
	PTW	1.00 (107)	0 (0)	0 (0)	1.000	0.000	0.000
	LC	0 (0)	0.11 (12)	0.89 (95)	0.056	0.944	0.100
	LS	0.78 (83)	0.21 (22)	0.01 (1)	0.887	0.113	0.181

g.39807T>G		TT	TG	GG	T	G	
	PTB	0 (0)	0.03 (3)	0.97 (109)	0.013	0.987	0.026
	PTW	0.79 (85)	0.19 (20)	0.02 (2)	0.888	0.112	0.179
	LC	0.02 (2)	0 (0)	0.98 (105)	0.019	0.981	0.036
	LS	0 (0)	0.01 (1)	0.99 (105)	0.005	0.995	0.009

g.40862G>A		GG	GA	AA	G	A	
	PTB	0 (0)	0.02 (2)	0.98 (110)	0.009	0.991	0.018
	PTW	0.86 (92)	0.12 (13)	0.02 (2)	0.921	0.079	0.136
	LC	0 (0)	0 (0)	1.00 (107)	0.000	1.000	0.000
	LS	0 (0)	0.17 (18)	0.83 (88)	0.085	0.915	0.143

*Note*. PTB means Putian Black duck (*N* = 112), PTW means Putian White duck (*N* = 107), LC means Liancheng White duck (*N* = 107), and LS means Longsheng Jade-green duck (*N* = 106). PIC means polymorphism information content. PIC < 0.25 indicates a low level of polymorphism, 0.25 < PIC < 0.5 indicates a medium level of polymorphism, and PIC > 0.5 indicates a high level of polymorphism.

## Data Availability

The data used to support the findings of this study are available from the corresponding author upon request.

## Supplementary Materials

Supplementary Materials Supplementary Figure 1: bioinformatics analysis of the CpG island of the duck *MITF* gene. The blue region is the CpG island. Supplementary Figure 2: PCR-RFLP genotyping patterns of four SNPs in the duck *MITF* gene. Supplementary Table 1: primer pairs used for identifying the polymorphisms of the *MITF* gene.
