# Supplementary material for: Integrated Analysis of mRNA Expression, CpG Island Methylation, and Polymorphisms in the MITF Gene in Ducks (Anas platyrhynchos)
Source: Biomed Res Int. 2019 Sep 23;2019:8512467. doi: 10.1155/2019/8512467 (PMC6778931; doi:10.1155/2019/8512467)
Supplement: Supplementary Materials — Supplementary Figure 1: bioinformatics analysis of the CpG island of the duck MITF gene. The blue region is the CpG island. Supplementary Figure 2: PCR-RFLP genotyping patterns of four SNPs in the duck MITF gene. Supplementary Table 1: primer pairs used for identifying the polymorphisms of the MITF gene. [file 8512467.f1.pdf]

Supplementary Materials

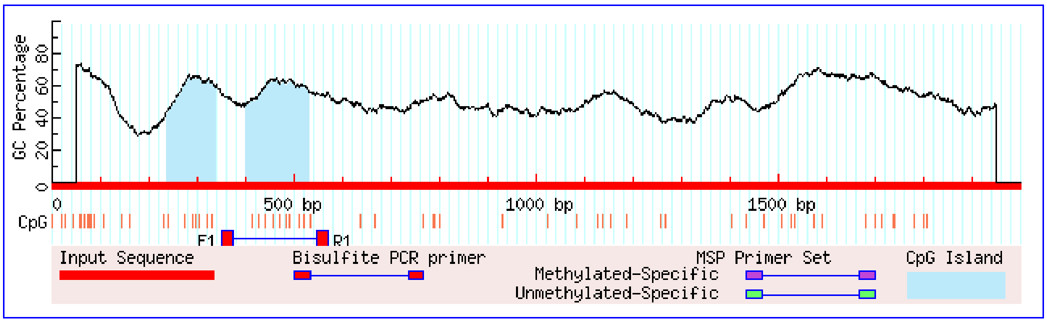

Supplementary Figure 1: Bioinformatics analysis of the CpG island of duck *MITF* gene. The blue region is the CpG island.

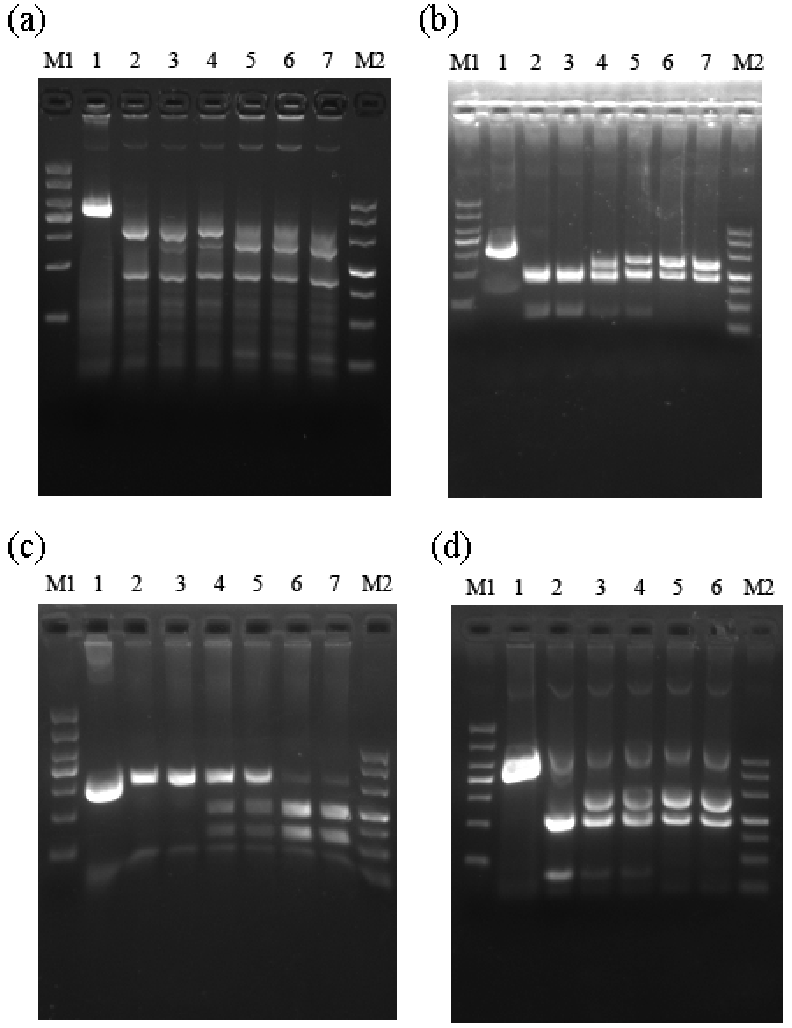

Supplementary Figure 2: PCR-RFLP genotyping patterns of four SNPs in duck *MITF* gene.

(a) PCR-*Hae*II-RFLP analysis for the g.312C>T site; the PCR products were digested with the *Hae*II enzyme and cleaved into 60-, 168-, and 257-bp fragments for the C allele and 168- and 317-bp fragments for the T allele. Lane 1, PCR product; lane 2, TT genotype; lanes 3 to 4, CT genotype; lanes 5 to 7, CC genotype. (b) PCR-*Hae*III-RFLP analysis for the g.32813G>A site; the digestion of a 457-bp DNA fragment with the *Hae*III enzyme produced fragments of 75-, 184-, and 198- bp for the G allele and fragments of 198- and 259- bp for the A allele. Lane 1, PCR product; lanes 2 to 3, GG genotype; lanes 4 to 5, GA genotype; lanes 6 to 7, AA genotype. (c) PCR-*Apo*I-RFLP analysis for the g.39807T>G site; a 329-bp fragment for the G allele and 128-

and 201-bp fragments for the T allele when digested with the *Apo*I enzyme. Lane 1, PCR product; lanes 2 to 3, GG genotype; lanes 4 to 5, TG genotype; lanes 6 to 7 TT genotype. (d) PCR-*Hin*fI-RFLP analysis for the g.40862G>A site; the digestion of the PCR product with the *Hin*fI enzyme resulted in fragments of 223- and 317- bp for the G allele and fragments of 80-, 223-, and 237- bp for the A allele. Lane 1, PCR product; lane 2, AA genotype; lanes 3 to 4, GA genotype; lanes 5 to 6, GG genotype. M1, DL1000 DNA marker; M2: DL500 DNA marker.

Supplementary Table 1: Primer pairs used for identifying the polymorphisms of *MITF* gene

Primer name	Primer sequence (5'-3')	Product size (bp）	Tm (°C)
GF1	TTCTCGTGGATGGTGGCTGTAT	1140	57.1
GR1	TTATGTGGACTTACCCTGTGC		
GF2	AGCCCTTTCCTAACTCCT	568	56.6
GR2	CAACTATTCACTGCGTGCATCA		
GF3	TTGTACGGGCACCGATAT	457	52.6
GR3	CTTGAGGAGCAGCGATTA		
GF4	TGAATAAGCCACTCCGTCTA	1229	54.5
GR4	CAAACACTTCTACTGCCACA		
GF5	TCGCCCAGACAGCAAGTAATA	1090	55.2
GR5	TAAGGAAACCCACAAGCAAAA		
GF6	TCGTGTCTGGGTAGGTTC	880	56.3
GR6	TTGGAGGCATTGGTAGTT		
